# Autism Spectrum Disorder as Early Neurodevelopmental Disorder: Evidence from the Brain Imaging Abnormalities in 2–3 Years Old Toddlers

**DOI:** 10.1007/s10803-014-2033-x

**Published:** 2014-01-14

**Authors:** Zhou Xiao, Ting Qiu, Xiaoyan Ke, Xiang Xiao, Ting Xiao, Fengjing Liang, Bing Zou, Haiqing Huang, Hui Fang, Kangkang Chu, Jiuping Zhang, Yijun Liu

**Affiliations:** 1 Child Mental Health Research Center, Nanjing Brain Hospital Affiliated of Nanjing Medical University, Nanjing GuangZhou Road 264#, Nanjing, 210029 China; 2 Department of Psychiatry, McKnight Brain Institute, University of Florida, Gainesville, FL 32601 USA

**Keywords:** Autism spectrum disorder, Toddler, Magnetic resonance imaging, Voxel based morphometry, Diffusion tensor imaging

## Abstract

Autism spectrum disorder (ASD) is a complex neurodevelopmental condition that occurs within the first 3 years of life, which is marked by social skills and communication deficits along with stereotyped repetitive behavior. Although great efforts have been made to clarify the underlying neuroanatomical abnormalities and brain-behavior relationships in adolescents and adults with ASD, literature is still limited in information about the neurobiology of ASD in the early age of life. Brain images of 50 toddlers with ASD and 28 age, gender, and developmental quotient matched toddlers with developmental delay (DD) (control group) between ages 2 and 3 years were captured using combined magnetic resonance-based structural imaging and diffusion tensor imaging (DTI). Structural magnetic resonance imaging was applied to assess overall gray matter (GM) and white matter (WM) volumes, and regional alterations were assessed by voxel-based morphometry. DTI was used to investigate the white matter tract integrity. Compared with DD, significant increases were observed in ASD, primarily in global GM and WM volumes and in right superior temporal gyrus regional GM and WM volumes. Higher fractional anisotropy value was also observed in the corpus callosum, posterior cingulate cortex, and limbic lobes of ASD. The converging findings of structural and white matter abnormalities in ASD suggest that alterations in neural-anatomy of different brain regions may be involved in behavioral and cognitive deficits associated with ASD, especially in an early age of 2–3 years old toddlers.

## Introduction

Autism spectrum disorder (ASD) is a complex neurodevelopmental disorder characterized by impairments in social interaction and communication that begin at a young age, which commonly exhibits repetitive behaviors and restricted interests. Currently, ASD occurs in as much as 0.6 % of the global population (Elsabbagh et al. 2012), and is more common in males (Baron-Cohen et al. 2011; Baird et al. 2006). There is increasing evidence that the heterogeneity and complexity of core symptoms in ASD can be explained as a fundamental impairment in both grey and white matter structure (Minshew and Williams 2007). In this perspective, a recent noninvasive magnetic resonance-based method of combined structure imaging and diffusion tensor imaging (DTI) specifically sensitive to the presence of brain structure and WM integrity alterations, has allowed for a better understanding of the neuroanatomical abnormalities in ASD.

Although the etiology of ASD remains unclear, a growing number of efforts have been made on studies of the neural bases in ASD. There was variability in the literature on brain imaging findings in ASD. Early brain overgrowth was probably the most replicated finding in this population (Bryńska 2012; Courchesne et al. 2003). There were some of the available evidence of both grey matter (GM) and WM volume abnormalities in ASD group compare to normal people (Courchesne et al. 2001; Ulay and Ertuğrul 2009). Additionally some specific brain regions were particularly implicated, including the frontal lobe, temporal lobe, limbic structure, amygdale, basal ganglia and cerebellar regions (Carper et al. 2002; Hazlett et al. 2005; Nordahl et al. 2012; Rojas et al. 2006; Schumann et al. 2010; Ulay and Ertuğrul 2009). Moreover, few papers based on DTI have shed light on microstructural WM integrity in certain brain regions in ASD (Aoki et al. 2013; Barnea et al. 2004).Despite these existing findings, it’s still difficult to compare those reports with each other, depending on substantial differences not only as to the clinical and demographic characteristics of the samples, but also as to the methods of analysis. To date, as far as we know, few studies have combined neuroimaging data from DTI and structural imaging with voxel-based morphometry (VBM) to investigate the neuroanatomical changes in 2–3 years old toddlers with ASD. The goal of current research is to investigate the available brain imaging data and examine their implication for understanding the neuroanatomical bases of ASD. In order to assess these changes, the whole brain volumes, regional anatomy of GM and WM, and WM integrity in 2–3 years old toddlers with ASD and other DD controls were measured using magnetic resonance imaging (MRI) with VBM and DTI. In particular, the purpose of this research is twofold: first, to examine possible brain imaging abnormalities in the structure and WM integrity between ASD children and DD controls; second, to analyze voxel-based brain volume differences between ASD and DD group.

## Methods

### Participants and Assessments

A total of 50 toddlers with ASD (mean age 29.92 ± 5.54 months) and 28 age, gender, and developmental quotient (DQ) matched toddlers with DD (mean age 28.25 ± 4.38 months) were included in a case–control study conducted from May 2010 to November 2011 at the Child Mental Health Research Center of the Nanjing Brain Hospital affiliated of Nanjing Medical University. The study protocol was fully disclosed to all participants and their guardians, and written informed consent was obtained from each participant’s guardian according the provision of the Declaration of Helsinki. The study protocol was approved by the Institutional Review Board of the Nanjing Brain Hospital affiliated of Nanjing Medical University.

The diagnoses of ASD were based on the DSM-IV-TR criteria of pervasive developmental disorders (Rett’s Syndrome and Heller’s Syndrome was excluded) by two licensed child psychiatrists. All patients were assessed by childhood autism rating scale (CARS) (Schopler et al. 1980) and autism diagnostic inventory-revised (ADI-R) (Lord et al. 1997). The inclusion criteria of DD was mental retardation or communication disorders on DSM-IV-TR. Mental retardation caused by chromosome abnormalities or gene defect s such as: Down syndrome, Klinefelter’s syndrome, Fragile X syndrome, congenital hypothyroidism, Phenylketonuria and Prader–Willi syndrome was excluded from DD group by *genetic tests.* We also excluded patients who had a history of head injury, neurological disorders, or other major medical problems from ASD and DD group. DQ scores of all participants were obtained using the Bayley Scales of Infant Development—Chinese Version (BSID-C) (Shou et al. 1993).

### Imaging Data Acquisition

Structural MRI images were acquired using a standard quadrature head coil on a 3.0 T Verio MRI system (Siemens Medical Systems, Germany). Before MRI scanning, each subject was sedated by using chloral hydrate with parental consent. All the participants took chloral hydrate (0.5 g in 10 ml) for oral administration around 12 ml each time (50 mg/kg). The maximum dosage was usually <20 ml. About 85 % of the subjects successfully took chloral hydrate for oral administration at first time, and the rest took enema instead at next time. So each participant was sedated before MRI scanning.

During scanning, the patient’s head was gently restrained by foam cushions. High-resolution MRI images for volumetric analysis were obtained with a T1-weighted (T1W) three-dimensional (3D) spoiled gradient (SPGR) sequence with the following parameters: TR = 2,530 ms, TE = 3.34 ms, flip angle = 7°, field of view (FOV) = 256 mm × 256 mm^2^, in-plane resolution = 256 × 192, inversion time = 1,100 ms, and slice thickness = 1.33 mm. Image orientation parallel to the anterior commissure–posterior commissure (AC–PC) plane was used.

Diffusion tensor imaging (DTI) were obtained with single shot echo planar (SE-EPI) sequences with diffusion gradients applied in 30 non-collinear directions and b = 1,000 s/mm^2^. The thickness of each slice was 2.5 mm without gap. The sequence parameters for DTI were: TE = 104 ms; repetition time (TR) = 9,000 ms; FOV = 230 × 230 mm^2^, and acquisition matrix = 128 × 128. Total DTI scanning time was 5.1 min. Routine clinical MRI scans (T1W, T2W, and fluid-attenuated inversion recovery) were conducted to further reveal incidental pathological abnormalities.

### Analysis of Structural Images

All T1W and T2W MRI scans were assessed by a neuroradiologist to exclude macroscopic pathology, WM hyperintensity, and abnormal ventricular/sulcal enlargement by age. Structural MRI was used to compare global and regional total brain volume, GM, and WM variations. All images were processed with SPM8 (http://www.fil.ion.ucl.ac.uk/spm) and VBM8 Toolbox (http://dbm.neuro.uni-jena.de/vbm.html).

Customized template was created based on all participants during the optimized VBM8 protocol, which was then used as a prior image to normalize, segment and modulate all images. Briefly, T1W MRI images were bias field-corrected and GM, WM, and cerebrospinal fluid (CSF) were identified. Global GM, WM, CSF, and total intracranial volume (TIV) were then calculated in native space for each subject and used for between-group comparisons. Individual native space GM and WM segments were normalized with an affine registration. From these affine-registered GM and WM segments, an average diffeomorphic anatomical registration through exponentiated lie algebra (DARTEL) template of all subjects in Montreal Neurological Institute (MNI) space was constructed. Next, affine-registered GM segments were warped against the average template using high-dimensional DARTEL means and modulated. Resultant GM maps code the relative local volume of GM in MNI space (Chapman et al. 2006). To distinguish possible artifacts and failed segmentation or normalization, a quality check was performed using the SPM “check reg” function and MRIcron (http://www.cabiatl.com/mricro/mricron/index.html). When no unexpected abnormalities were reported, GM maps were smoothed with an 8 × 8 × 8 mm^3^ full-width-at-half-maximal kernel.

### Analysis of Diffusion Tensor Images

SPM8, DTI studio 2.4.01 (https://www.mristudio.org/), and a custom-developed in-house software system based on MatLab 2009 (http://www.mathworks.cn/) were used for preprocessing and analysis of DTI data. In short, data was denoised, and images were used to calculate FA and mean diffusion (MD) in DTI studio. Because stock templates may result in mistakes in FA and MD difference localization, a custom template for participants was created, and data was spatially normalized into this customized template. Resultant normalized maps were smoothed with 8 mm full-width-at-half-maximum isotropic Gaussian kernels for SPM8 analysis. Group analyses were carried out using random effects voxel-wise multiple regression analyses with a threshold at *t* > 3.20 (*p* < 0.001, uncorrected) and a spatial extent threshold of k = 50 voxels. These parameters were designed to remove isolated small regions of differences, allowing significantly different regions of interest (ROIs) in FA and MD to be clearly delineated. All coordinates were provided in MNI space.

### Statistical Analysis

Between-group differences in age, gender, DQ, CARS score, and ADI-R scores were calculated using *t* tests or *χ* ^2^ analyses. Global differences in whole brain structure were assessed by independent-sample *t* test in SPSS version 19.0 (IBM, USA). GM and WM volumes as well as total intracranial volume (TIV) were included as test variables, and diagnoses as ASD and DD were the primary grouping variables. Differences in regional GM and WM volumes as well as differences in FA and MD were thresholded at *p* value of <0.001 (two-tailed tests) uncorrected for multiple comparisons, with an extent threshold of 50 voxels to eliminate isolated small areas of volume differences using two-sample *t* tests in SPM8 (http://www.fil.ion.ucl.ac.uk/spm).

## Results

### Sample Characteristics

No significant differences were observed between ASD and DD (control group) by gender (*p* = 0.55), age (*p* = 0.17), or DQ (*p* = 0.07) (Table 1). However, ASD group had higher scores than control group in CARS and all ADI-R subtest (*p* < 0.01).

**Table 1:** Subjects’ demographic variables

	ASD	DD	*p* value^a^
Number of subjects	50	28	NA
Gender (male:female)	42:8	22:6	0.55
Age (m)	29.92 ± 5.54	28.25 ± 4.38	0.17
DQ^b^	68.00 ± 12.14	74.54 ± 15.76	0.07
CARS^c^	34.76 ± 4.32	28.61 ± 4.90	0.00**
ADI-R social^d^	21.26 ± 4.96	12.63 ± 6.07	0.00**
ADI-R communication^d^	12.04 ± 2.57	8.46 ± 3.85	0.00**
ADI-R behavior^d^	3.65 ± 2.16	1.89 ± 1.89	0.00**

** *p* < 0.01

^a^ *p* value for gender using *χ* ^2^ test; other *p* values for the comparison between ASD and DD are based on two-sample *t* tests

^b^Based on the Bayley scales of infant development (BSID)

^c^Based on the childhood autism rating scale (CARS)

^d^Based on the autism diagnostic inventory-revised (ADI-R)

### Global Volumetric Measurements

Because participants were well-matched, global volumetric differences were determined by two-sample *t* tests. Global GM and WM volumes for ASD and control group were shown in Table 2, indicating larger global GM and WM volumes in ASD (*p* = 0.048 and *p* = 0.047, respectively). Moreover, TIV comparisons indicated a non-significant higher trend for ASD compared with DD.

**Table 2:** Global volumetric measures in ASD and DD

	ASD	DD	*T*	*p* value
TIV (ml)	1,097.67 ± 106.74	1,044.44 ± 117.31	1.978	0.052
GM (ml)	737.39 ± 66.99	701.61 ± 83.32	2.011	0.048*
WM (ml)	360.28 ± 49.38	336.88 ± 41.78	2.021	0.047*

* *p* < 0.05

### Regional GM and WM Volumes

**Fig. 1:**
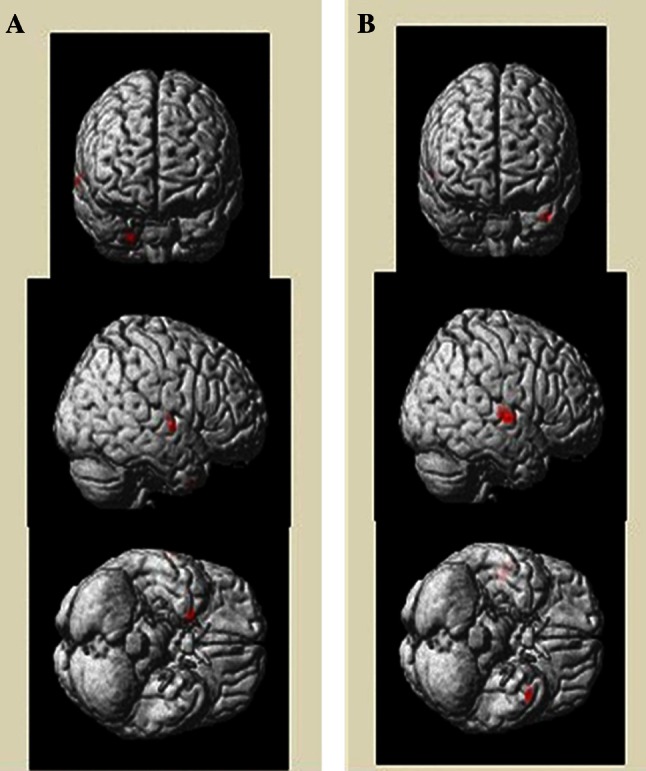
Mapping of the regional GM (**a**) and WM (**b**) differences between ASD patients and DD controls. The localization of regions showing significant difference (*p* < 0.001) were projected into a template created with the data from our participants output from the SPM8. The anatomical locations of regions were listed in Table 3A, B for GM and WM differences respectively. *L* left, *R* right, *A* anterior, *P* posterior

In addition to global volumetric differences, regional GM and WM volumes also differed between ASD and control group (Table 3A, B), revealing that more GM volumes were localized in the right superior temporal gyrus of the temporal lobe in ASD (Fig. 1a). Conversely, greater WM volumes were observed in ASD in the right superior temporal gyrus, left middle temporal gyrus, right insula, and right Heschl’s gyrus (Fig. 1b). Notably, no significant decreases in GM and WM volumes were observed in any regions between ASD and control group.

**Table 3:** Significant neuroanatomical differences between ASD and DD

Localization of the regions with significant neuroanatomical differences	K^a^	*T*	Talairach coordinates of the voxel with the maximal significance		
			x	y	z
*A: GM volumes*					
ASD > DD					
Superior temporal gyrus, brodmann area 22 (R)	66	3.50	52	−10	5
Temporal lobe, brodmann area 38 (R)	50	3.38	24	9	−42
*B: WM volumes*					
ASD > DD					
Temporal lobe, superior temporal gyrus (R)	260	4.18	56	−12	0
Temporal lobe, middle temporal gyrus (L)	88	3.97	−44	8	−32
Insula (R)	73	3.75	47	−15	5
Heschl (R)	63	3.47	35	−19	9
*C: FA*					
ASD > DD					
Corpus callosum, posterior cingulate, limbic lobe	177	4.18	4	−38	20
*D: MD*					
ASD < DD					
Posterior cingulate, corpus callosum, limbic lobe (L)	132	4.29	−12	−44	26
Insula (L)	138	3.78	−30	−16	22

^a^Spatial extent (number of voxels) of the brain region showing significant increases in gray matter volume at a threshold of *t* > 3.20 (*p* < 0.001, and cluster size *k* = 50 voxels)

### Local WM FA and MD

**Fig. 2:**
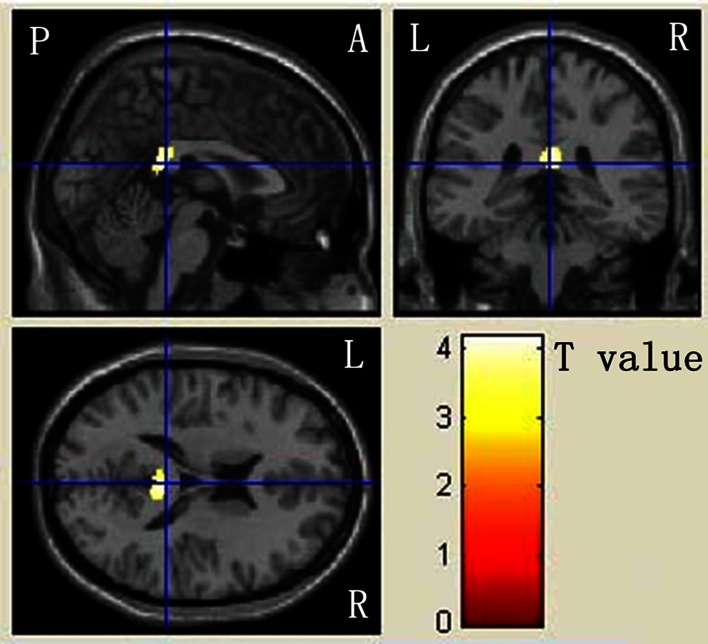
Mapping of FA differences between ASD patients and DD controls. The localization of regions showing significant difference (*p* < 0.001) were projected into a template created with the data from our participants output from the SPM8. The anatomical locations of regions were listed in Table 3C. The regions that are increased for ASD than DD are presented in *red* to *yellow*. *L* left, *R* right, *A* anterior, *P* posterior

**Fig. 3:**
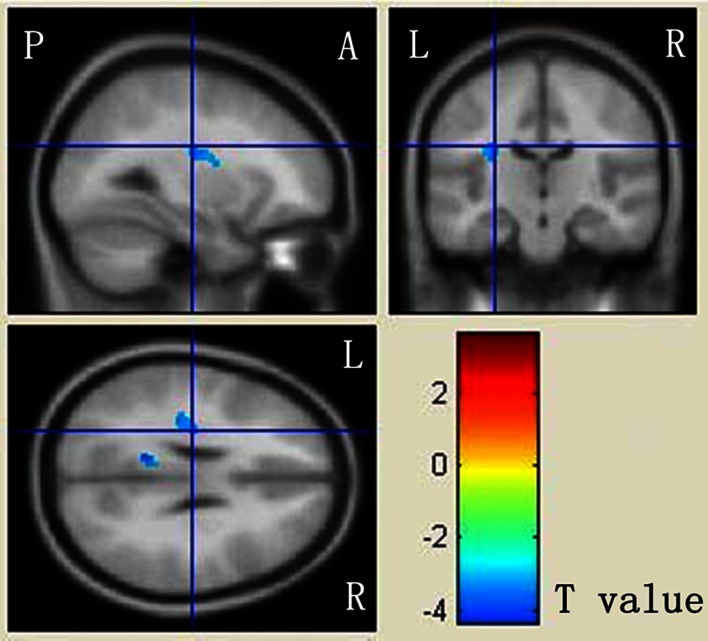
Mapping of MD differences between ASD patients and DD controls. The localization of regions showing significant difference (*p* < 0.001) were projected into a template created with the data from our participants output from the SPM8. The anatomical locations of regions were listed in Table 3D. The regions that are decreased for ASD than DD are presented in *yellow* to *blue*. *L* left, *R* right, *A* anterior, *P* posterior

Voxel-based analysis was performed to determine the brain WM microstructure of ASD, a characteristic primarily caused by high FA and low MD in some cerebral regions (Huster et al. 2009; Menzler et al. 2011; Westerhausen et al. 2011) (Table 3C, D). ASD strongly exhibited higher FA in the CC, posterior cingulate cortex, and limbic lobe (Fig. 2). In addition, ASD exhibited lower MD in left CC, posterior cingulate, limbic lobe, and insular cortex (Fig. 3). Similarly, no significantly lower FA or higher MD values were observed in ASD or control group.

## Discussion

In this study, brain structure anatomy and WM integrity in ASD toddlers were investigated following a combined approach of structure imaging and DTI. The neurophysiological results provided three important findings: (1) These young ASD children possessed larger global GM and WM volumes than DD group of the same ages, genders and DQ scores; (2) these ASD children exhibited elevated GM volumes most commonly observed in the right superior temporal gyrus of the temporal lobe and elevated WM volumes most commonly observed in the right superior temporal gyrus, left middle temporal gyrus, right insular cortex, and right Heschl’s gyrus; (3) ASD also exhibited higher FA and lower MD in the CC, posterior cingulate cortex, and limbic lobe as well as lower MD in the insular cortex.

The first result not only agreed with the initial evidence of brain and cerebral enlargement in toddlers with AS reported by Courchesne and his colleagues in 2001, but also extends the stage into Chinese population at which total brain volumes were increased in ASD patients at early age. Courchesne et al. (2001) indicated that by age 2–3 years over 90 % of autistic toddlers exhibited abnormally larger total brain, and the autistic 2- to 3-year-olds had more cerebral (18 %) and cerebellar (39 %) WM, and more cerebral cortical GM (12 %) than normal boys. Furthermore, similar findings were described in a recent study concerning brain WM volumes in autistic boys of ASD which also suggested that there was an overall increase in brain volumes compared with controls (Herbert et al. 2003). The current study demonstrated results consistent with a large body of literature supporting that possible brain imaging abnormalities in the structure indeed existed in ASD children at the early age (Courchesne et al. 2001; Cosgrove et al. 2007; Hazlett et al. 2005; Schumann et al. 2010; Sparks et al. 2002).

Secondly, greater regional GM volumes in the right superior temporal gyrus and higher WM volumes in the right superior temporal gyrus, left middle temporal gyrus, right insular cortex, and right Heschl’s gyrus were observed in ASD toddlers in the current study by using optimized VBM. Several previous studies performed on ASD also reported that autistic children exhibited extreme enlargement of GM and WM volumes in the frontal and temporal lobes, a slightly variant result from many other studies (Carper et al. 2002; Cosgrove et al. 2007; Courchesne et al. 2004; Hazlett et al. 2005; Rojas et al. 2006; Schumann et al. 2010). Notably, the structure found in our report were predominantly localized in the temporal regions, which were implicated in social perception, language, and “theory-of-mind”, an ability to put oneself into someone else’s shoes, to imagine their thoughts and feelings, all of which were impaired in ASD. In accordance with above findings, we confirmed the GM and WM volumes increase in the temporal regions of 2- to 3-year-old toddlers with ASD.

Diffusion tensor imaging (DTI) data from the current study also demonstrated that changes in microstructural WM organization with increased FA and reduced MD were apparent in the CC and cingulum of the ASD group. The CC is the largest inter-hemispheric tract in the brain and it is considered to be involved in emotion and social functioning, as well as in higher cognitive processes, such as decoding non-literal meaning, affective prosody, and understanding humour (Badaruddin et al. 2007; Brown et al. 2005a, b). The cingulate cortex participates in tasks related to higher-level cognitive processes, like empathic cognition, social behavior and pain perception in which is more likely to be hypoactivated in ASD (Di Martino et al. 2009; Thakkar et al. 2008). Most DTI researches on adolescents or adults with ASD showed reduced FA in several brain regions, including the CC and cingulum (Alexander et al. 2007; Keller et al. 2007; Weinstein et al. 2011).

However, in a study (Cheng et al. 2010) carried out on adolescents with ASD, an imbalance of FA was found, as some areas showed an increased FA, while others a decreased FA. Moreover, Billeci et al. (2012) found an increased FA in about 5 years old ASD in numerous WM tracts like CC and cingulum. These contradictory results could reflect a change of the trend of FA in young children compared with adolescents and adults. Furthermore, it may be linked to the alteration in ASD brain volume, which has a macrostructural expansion in the first years of life and then shifts to an abnormally slow growth (Jones and Cercignani 2010; Liu et al. 2010; Muratori et al. 2012; Vos et al. 2011; Westerhausen et al. 2011). In fact, the weak concordance among DTI investigations largely depends on the wide heterogeneity both of ASD clinical phenotype and of methods of analysis, which makes it more difficult for comparison of results. Furthermore, experimental artifacts and confounding factors should be taken into consideration in order to enhance the reliability and comparability of DTI results (Walker et al. 2012). Caution is needed in interpreting high FA levels, because this change could represent an index of various WM microstructural alterations, like increased myelination, axon size and density, path geometry, and the presence of crossing fiber pathway. To date, as our knowledge, the voxel-based analysis has been applied to limited number of ASD studies on young autistic children, especially in this early stage of 2- to 3-year-old toddlers.

The results of this study should be considered in lieu of the matching strategy, which involved the use of DD group as controls, a group which may not accurately reflect characteristics of the general population. ASD commonly co-occurs with other neurodevelopmental disorders. Many ASD individuals have language deficits, ranging from complete lack of speech through language delays, poor comprehension of speech, or stilted and overly literal language. So it is impossible to exclude the language impairment in our ASD group. Specific learning disabilities are also common in individuals with an ASD. But it is difficult to identify the specific learning disorder during 2–3 years old. Thus, further studies with larger and more diverse cohorts will be required to verify these findings. Furthermore, when comparing the results of this relatively small study with much larger studies without adjustment, it’s impossible to avoid errors. Although customized template was created based on all participants, the use of VBM was still unable to overcome the problem of technical difficulties for segmentation at this early age. Lastly, not including the autism diagnostic observation schedule (ADOS) as part of the diagnostic process should be included as a limitation of our study. Therefore, further substantiation of brain regions, especially for microstructural organization, remains necessary to confirm these findings.

## Conclusions

Notwithstanding the problematic issues raised above, this study adds to the limited existing literature on early age of children with ASD. Several GM and WM abnormalities in brain structure and an increase of FA linked to ASD behavioral deficits (e.g. temporal lobes, CC and cingulum) were detected. These results provide powerful evidence to support that 2- to 3-year-old toddlers with ASD exhibit neuro-imaging abnormalities of certain brain regions and present a link between previous behavioral findings and neuroanatomical features in a straightforward manner. Further research will be addressed to confirm and apply these observations in ASD of variant age and demographics.

## References

[CR1] Alexander AL, Lee JE, Lazar M, Boudos R, DuBray MB, Oakes TR, Miller JN, Lu J, Jeong EK, McMahon WM, Bigler ED, Lainhart JE (2007). Diffusion tensor imaging of the corpus callosum in autism. Neuroimage.

[CR4] Aoki Y, Abe O, Nippashi Y, Yamasue H (2013). Comparison of white matter integrity between autism spectrum disorder subjects and typically developing individuals: A meta-analysis of diffusion tensor imaging tractography studies. Molecular Autism.

[CR6] Badaruddin DH, Andrews GL, Bölte S, Schilmoeller KJ, Schilmoeller G, Paul LK, Brown WS (2007). Social and behavioral problems of children with agenesis of the corpus callosum. Child Psychiatry and Human Development.

[CR7] Baird G, Simonoff E, Pickles A, Chandler S, Loucas T, Meldrum D, Charman T (2006). Prevalence of disorders of the autism spectrum in a population cohort of children in South Thames: The Special Needs and Autism Project (SNAP). Lancet.

[CR8] Barnea GN, Kwon H, Menon V, Eliez S, Lotspeich L, Reiss AL (2004). White matter structure in autism: Preliminary evidence from diffusion tensor imaging. Biological Psychiatry.

[CR14] Baron-Cohen S, Lombardo MV, Auyeung B, Ashwin E, Chakrabarti B, Knickmeyer R (2011). Why are autism spectrum conditions more prevalent in males?. PLoS Biology.

[CR19] Billeci L, Calderoni S, Tosetti M, Catani M, Muratori F (2012). White matter connectivity in children with autism spectrum disorders: A tract-based spatial statistics study. BMC Neurology.

[CR21] Brown WS, Paul LK, Symington M, Dietrich R (2005). Comprehension of humor in primary agenesis of the corpus callosum. Neuropsychologia.

[CR22] Brown WS, Symingtion M, VanLancker SD, Dietrich R, Paul LK (2005). Paralinguistic processing in children with callosal agenesis: Emergence of neurolinguistic deficits. Brain Language.

[CR23] Bryńska A (2012). Seeking the aetiology of autistic spectrum disorder. Part 1: Structural neuroimaging. Psychiatria Polska.

[CR24] Carper RA, Moses P, Tigue ZD, Courchesne E (2002). Cerebral lobes in autism: Early hyperplasia and abnormal age effects. Neuroimage.

[CR27] Chapman E, Baron-Cohen S, Auyeung B, Knickmeyer R, Taylor K, Hackett G (2006). Fetal testosterone and empathy: Evidence from the empathy quotient (EQ) and the “reading the mind in the eyes” test. Social Neuroscience.

[CR29] Cheng Y, Chou KH, Chen IY, Fan YT, Decety J, Lin CP (2010). Atypical development of white matter microstructure in adolescents with autism spectrum disorders. NeuroImage.

[CR30] Cosgrove KP, Mazure CM, Staley JK (2007). Evolving knowledge of sex differences brain structure, function, and chemistry. Biological Psychiatry.

[CR31] Courchesne E, Carper R, Akshoomoff N (2003). Evidence of brain overgrowth in the first year of life in autism. American Medical Association.

[CR32] Courchesne E, Karns CM, Davis HR, Ziccardi R, Carper RA, Tigue ZD, Chisum HJ, Moses P, Pierce K, Lord C, Lincoln AJ, Pizzo S, Schreibman L, Haas RH, Akshoomoff NA, Courchesne RY (2001). Unusual brain growth patterns in early life in patients with autistic disorder: an MRI study. Neurology.

[CR33] Courchesne E, Redcay E, Kennedy DP (2004). The autistic brain: Birth through adulthood. Current Opinion in Neurology.

[CR34] Di Martino A, Ross K, Uddin LQ, Sklar AB, Castellanos FX, Milham MP (2009). Functional brain correlates of social and nonsocial processes in autism spectrum disorders: An activation likelihood estimation meta-analysis. Biological Psychiatry.

[CR35] Elsabbagh M, Divan G, Koh YJ, Kim YS, Kauchali S, Marcín C, Montiel-Nava C, Patel V, Paula CS, Wang C, Yasamy MT, Fombonne E (2012). Global prevalence of autism and other pervasive developmental disorders. Autism Research.

[CR36] Falter CM, Plaisted KC, Davis G (2008). Visuo-spatial processing in autism—testing the predictions of extreme male brain theory. Journal of Autism and Developmental Disorders.

[CR37] Gillberg C, Cederlund M, Lamberg K, Zeijlon L (2006). Brief report: ‘‘the autism epidemic’’. The registered prevalence of autism in a Swedish urban area. Journal of Autism and Developmental Disorders.

[CR38] Good CD, Johnsrude I, Ashburner J, Henson RN, Friston KJ, Frackowiak RS (2001). Cerebral asymmetry and the effects of sex and handedness on brain structure: A voxel-based morphometric analysis of 465 normal adult human brains. Neuroimage.

[CR39] Hazlett HC, Poe M, Gerig G, Smith RG, Provenzale J, Ross A, Gilmore J, Piven J (2005). Magnetic resonance imaging and head circumference study of brain size in autism: Birth through age 2 years. Archives of General Psychiatry.

[CR40] Herbert MR, Ziegler DA, Deutsch CK, O’Brien LM, Lange N, Bakardjiev A, Hodgson J, Adrien KT, Steele S, Makris N, Kennedy D, Harris GJ, Caviness VS (2003). Dissociations of cerebral cortex, subcortical and cerebral white matter volumes in autistic boys. Brain.

[CR41] Huster RJ, Westerhausen R, Kreuder F, Schweiger E, Wittling W (2009). Hemispheric and gender related differences in the midcingulum bundle: A DTI study. Human Brain Mapping.

[CR42] Inano S, Takao H, Hayashi N, Abe O, Ohtomo K (2011). Effects of age and gender on white matter integrity. American Journal of Neuroradiology.

[CR43] Jones DK, Cercignani M (2010). Twenty-five pitfalls in the analysis of diffusion MRI data. NMR in Biomedicine.

[CR44] Keller TA, Kana RK, Just MA (2007). A developmental study of the structural integrity of white matter in autism. NeuroReport.

[CR45] Knickmeyer RC, Baron-Cohen S (2006). Fetal testosterone and sex differences in typical social development and in autism. Journal of Child Neurology.

[CR46] Liu F, Vidarsson L, Winter JD, Tran H, Kassner A (2010). Sex differences in the human corpus callosum microstructure: A combined T_2_ myelin-water and diffusion tensor magnetic resonance imaging study. Brain Research.

[CR47] Lord C, Pickles A, McLennan J, Rutter M, Bregman J, Folstein S, Fombonne E, Leboyer M, Minshew N (1997). Diagnosing autism: Analyses of data from the Autism Diagnostic Interview. Journal of Autism and Developmental Disorders.

[CR48] Luders E, Narr KL, Thompson PM, Woods RP, Rex DE, Jancke L, Steinmetz H, Toga AW (2005). Mapping cortical gray matter in the young adult brain: Effects of gender. Neuroimage.

[CR49] Menzler K, Belke M, Wehrmann E, Krakow K, Lengler U, Jansen A, Hamer HM, Oertel WH, Rosenow F, Knake S (2011). Men and women are different: Diffusion tensor imaging reveals sexual dimorphism in the microstructure of the thalamus, corpus callosum and cingulum. Neuroimage.

[CR50] Minshew NJ, Williams DL (2007). The new neurobiology of autism: Cortex, connectivity and neuronal organization. Arch Neurology.

[CR51] Muratori F, Calderoni S, Apicella F, Filippi T, Santocchi E, Calugi S, Cosenza A, Tancredi R, Narzisi A (2012). Tracing back to the onset of abnormal head circumference growth in Italian children with autism spectrum disorder. Research of Autism Spectrum Disorder.

[CR52] Nordahl CW, Scholz R, Yang X, Buonocore MH, Simon T, Rogers S, Amaral DG (2012). Increased rate of amygdala growth in children aged 2 to 4 years with autism spectrum disorders: A longitudinal study. Archives of General Psychiatry.

[CR53] Oh JS, Song IC, Lee JS, Kang H, Park KS, Kang E, Lee DS (2007). Tractography-guided statistics (TGIS) in diffusion tensor imaging for the detection of gender difference of fiber integrity in the midsagittal and parasagittal corpora callosa. Neuroimage.

[CR54] Rojas DC, Peterson E, Winterrowd E, Reite ML, Rogers SJ, Tregellas JR (2006). Regional gray matter volumetric changes in autism associated with social and repetitive behavior symptoms. BMC Psychiatry.

[CR55] Schopler E, Reichler RJ, DeVellis RF, Daly K (1980). Toward objective classification of childhood autism: Childhood Autism Rating Scale (CARS). Journal of Autism and Developmental Disorders.

[CR56] Schumann CM, Bloss CS, Barnes CC, Wideman GM, Carper RA, Akshoomoff N, Pierce K, Hagler D, Schork N, Lord C, Courchesne E (2010). Longitudinal magnetic resonance imaging study of cortical development through early childhood in autism. Neuroscience.

[CR57] Schumann CM, Hamstra J, Goodlin-Jones BL, Lotspeich LJ, Kwon H, Buonocore MH, Lammers CR, Reiss AL, Amaral DG (2004). The amygdala is enlarged in children but not adolescents with autism; the hippocampus is enlarged at all ages. Neuroscience.

[CR58] Shou Y, Xue L, Zhi Y, Guo W (1993). The revision of the Bayley Scales of Infant Development (BSID) in China (city version). Chinese Journal of Clinical Psychology.

[CR59] Sparks BF, Friedman SD, Shaw DW, Aylward EH, Echelard D, Artru AA, Maravilla KR, Giedd JN, Munson J, Dawson G, Dager SR (2002). Brain structural abnormalities in toddlers with autism spectrum disorder. Neurology.

[CR60] Thakkar KN, Polli FE, Joseph RM, Tuch DS, Hadjikhani N, Barton JJ, Manoach DS (2008). Response monitoring, repetitive behaviour and anterior cingulate abnormalities in autism spectrum disorders (ASD). Brain.

[CR61] Ulay HT, Ertuğrul A (2009). Neuroimaging findings in autism: A brief review. Turk Psikiyatri Derg.

[CR62] Vos SB, Jones DK, Viergever MA, Leemans A (2011). Partial volume effect as a hidden covariate in DTI analyses. Neuroimage.

[CR63] Wakabayashi A, Baron-Cohen S, Uchiyama T, Yoshida Y, Kuroda M, Wheelwright S (2007). Empathizing and systemizing in adults with and without autism spectrum conditions: Cross-cultural stability. Journal of Autism and Developmental Disorders.

[CR64] Walker L, Gozzi M, Lenroot R, Thurm A, Behseta B, Swedo S, Pierpaoli C (2012). Diffusion tensor imaging in young children with autism: biological effects and potential confounds. Biology Psychiatry.

[CR65] Weinstein M, Ben Sira L, Levy Y, Zachor DA, Ben Itzhak E, Artzi M, Tarrasch R, Eksteine PM, Hendler T, Ben Bashat D (2011). Abnormal white matter integrity in young children with autism. Human Brain Mapping.

[CR66] Westerhausen R, Kompus K, Dramsdahl M, Falkenberg LE, Grüner R, Hjelmervik H, Specht K, Plessen K, Hugdahl K (2011). A critical re-examination of sexual dimorphism in the corpus callosum microstructure. Neuroimage.

[CR67] Wheelwright S, Baron-Cohen S, Goldenfeld N, Delaney J, Fine D, Smith R, Weil L, Wakabayashi A (2006). Predicting autism spectrum quotient (AQ) from the systemizing quotient-revised (SQ-R) and empathy quotient (EQ). Brain Research.

